# Publisher Correction to: TrancriptomeReconstructoR: data‑driven annotation of complex transcriptomes

**DOI:** 10.1186/s12859-021-04259-5

**Published:** 2021-07-15

**Authors:** Maxim Ivanov, Albin Sandelin, Sebastian Marquardt

**Affiliations:** 1 Department of Plant and Environmental Sciences, Copenhagen Plant Science Centre, University of Copenhagen, Thorvaldsensvej 40, 1871 Frederiskberg C, Denmark; 2 Department of Biology, University of Copenhagen, Ole Maaløes Vej 5, 2200 Copenhagen N, Denmark; 3 Biotech Research and Innovation Centre, University of Copenhagen, Ole Maaløes Vej 5, 2200 Copenhagen N, Denmark

## Publisher Correction to: BMC Bioinformatics (2021) 22:290 10.1186/s12859-021-04208-2

Following the publication of the original article [1], the authors identified missing additional files and references. The Additional files 1 and 2 including the references have been added to the original article.

The original article [1] has been corrected.

The publisher apologizes to the authors and readers for the inconvenience.

## Supplementary Information

**Additional file 1.** Vignette to TranscriptomeReconstructoR. The file describes an example pipeline for* de novo* calling of gene and transcript models by TranscriptomeReconstructoR, as well as detailed description of the algorithm.

**Additional file 2.** Supplementary Figures S1–S4.** Fig. S1**. Comparison of gene borders between TAIR10 and Araport11.** Fig. S2**. Metagene plot of PAS signal around TSS of sppRNA-containing genes.** Fig. S3**. Example of a novel gene encoding transient RNA.** Fig. S4**. Example of a gene misannotated in TAIR10 and Araport11.
