# Supplementary material for: TrancriptomeReconstructoR: data-driven annotation of complex transcriptomes
Source: BMC Bioinformatics. 2021 May 31;22:290. doi: 10.1186/s12859-021-04208-2 (PMC8166035; doi:10.1186/s12859-021-04208-2)
Supplement: Supplementary file 2 — Additional File 2. Supplementary Figures S1–S4. Fig. S1. Comparison of gene borders between TAIR10 and Araport11. Fig. S2. Metagene plot of PAS signal around TSS of sppRNA-containing genes. Fig. S3. Example of a novel gene encoding transient RNA. Fig. S4. Example of a gene misannotated in TAIR10 and Araport11. [file 12859_2021_4208_MOESM2_ESM.pdf]

Figure S1. Comparison of gene borders between TAIR10 and Araport11. Pairs of TAIR10 and Araport11 genes were matched by gene identifiers. The frequency polygon plot shows the distribution of differences (in bp) between 5' or 3' borders (left and right panels, respectively) of the matched gene pairs. A negative difference value means that the gene has a more upstream coordinate in Araport11 than in TAIR10. Similarly, a positive value means that the coordinate is downstream in Araport11 relative to TAIR10. The plot shows that Araport11 genes often start upstream and end downstream from their mates in TAIR10, i.e. are in general wider.

Figure S2. Metagene plot of PAS signal around TSS of sppRNA-containing genes. Triads of called genes, TAIR10 and Araport11 genes were matched by overlaps of genomic coordinates. The triads were subsetted to sppRNA-containing genes (n = 1326). Genomic windows (600 bp) were produced around the TSS positions. TIF-seq reads from the exosome mutant *hen2-2* sample were resized to their last bases (which correspond to the empirical PAS sites). The PAS signal was plotted along each of the 3 sets of genomic windows, and the metagene plots were overlaid. The vertical line corresponds to the TSS position. The average PAS signal plotted in TSS-centered windows predicted by the called genes (green) forms the most sharp peak at 100 bp downstream from the TSS. This means that 5' borders of the called genes are in a better agreement with the PAS sites of sppRNAs than 5' borders of the same genes in the existing annotations.

Figure S3. Example of a novel gene encoding transient RNA. Features on forward and reverse strands are shown in red and blue, respectively. The called transient RNA dubbed "Nascent_0037" is encoded on the forward strand. i.e. in antisense orientation to the AT1G05010 gene (ethylene-forming enzyme). This novel RNA transcript has no support from Nanopore Direct RNA-seq data (not shown). It was called from plaNET-seq data only, thus suggesting its transient and/or non-polyadenylated nature. The existence of this transcript was validated by two independent datasets (pNET-seq and chrRNA-seq) which are enriched for nascent RNA species. As expected, it is not visible in RNA-seq dataset, which explains why it is lacking from both TAIR10 and Araport11.

Figure S4. Example of a gene misannotated in TAIR10 and Araport11. Features on forward and reverse strands are shown in red and blue, respectively. The AT3G20270 (AtLBR-2) gene involved in pathogene response is annotated as a single gene in both TAIR10 and Araport11. However, TranscriptomeReconstructoR shows that it consists of two non-overlapping genes in tandem orientation. Both of these genes were classified as High Confidence, because they were supported by Nanopore Direct RNA-seq (not shown), CAGE-seq and PAT-seq data. The presence of two individual transcripts was further validated by independent TIF-seq data (the lowest track).

Figure S1

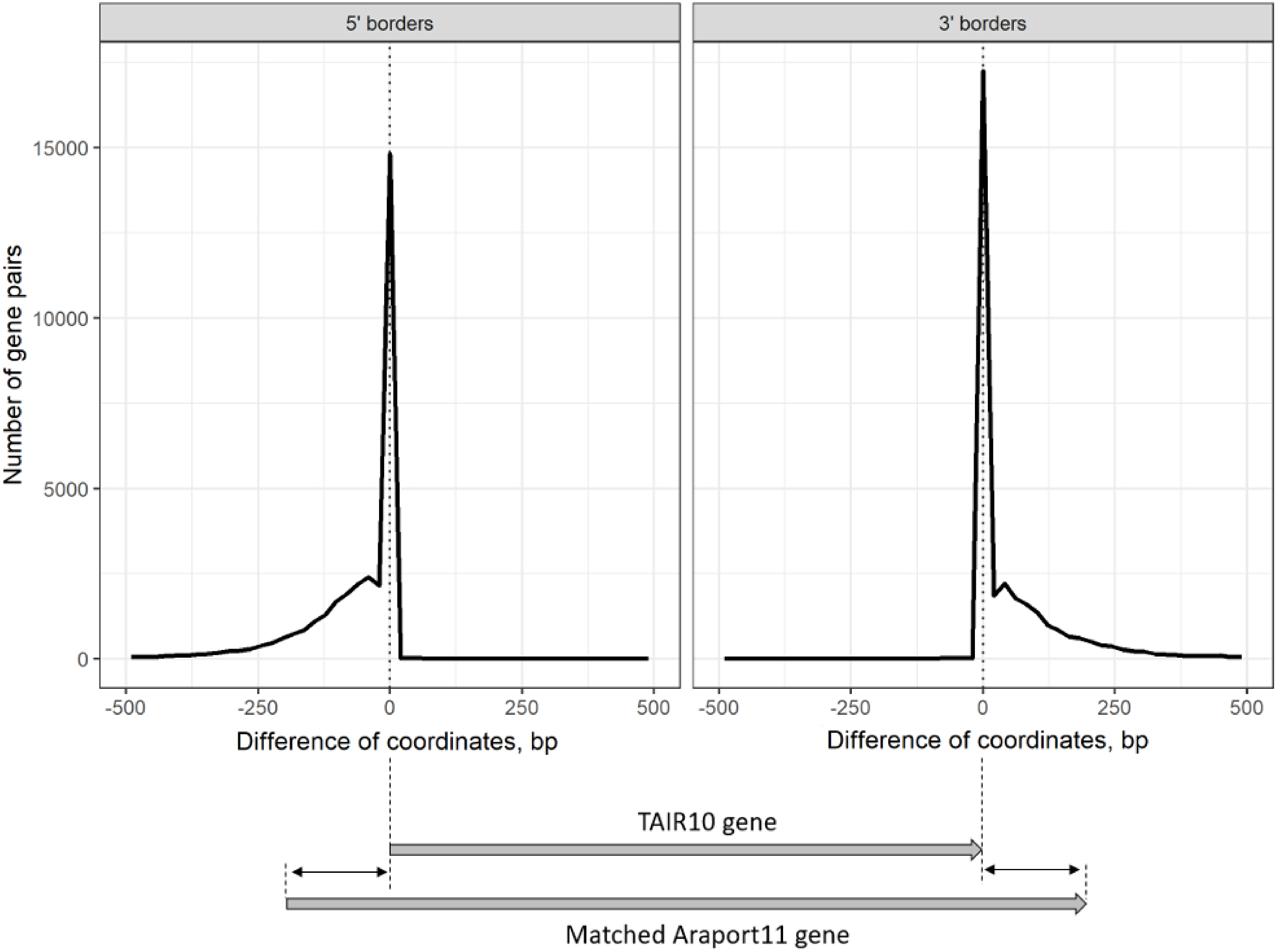

5' borders
3' borders
Number of gene pairs
15000
10000
5000
0
-500
-250
0
250
500
Difference of coordinates, bp
TAIR10 gene
Matched Araport11 gene

Figure S2

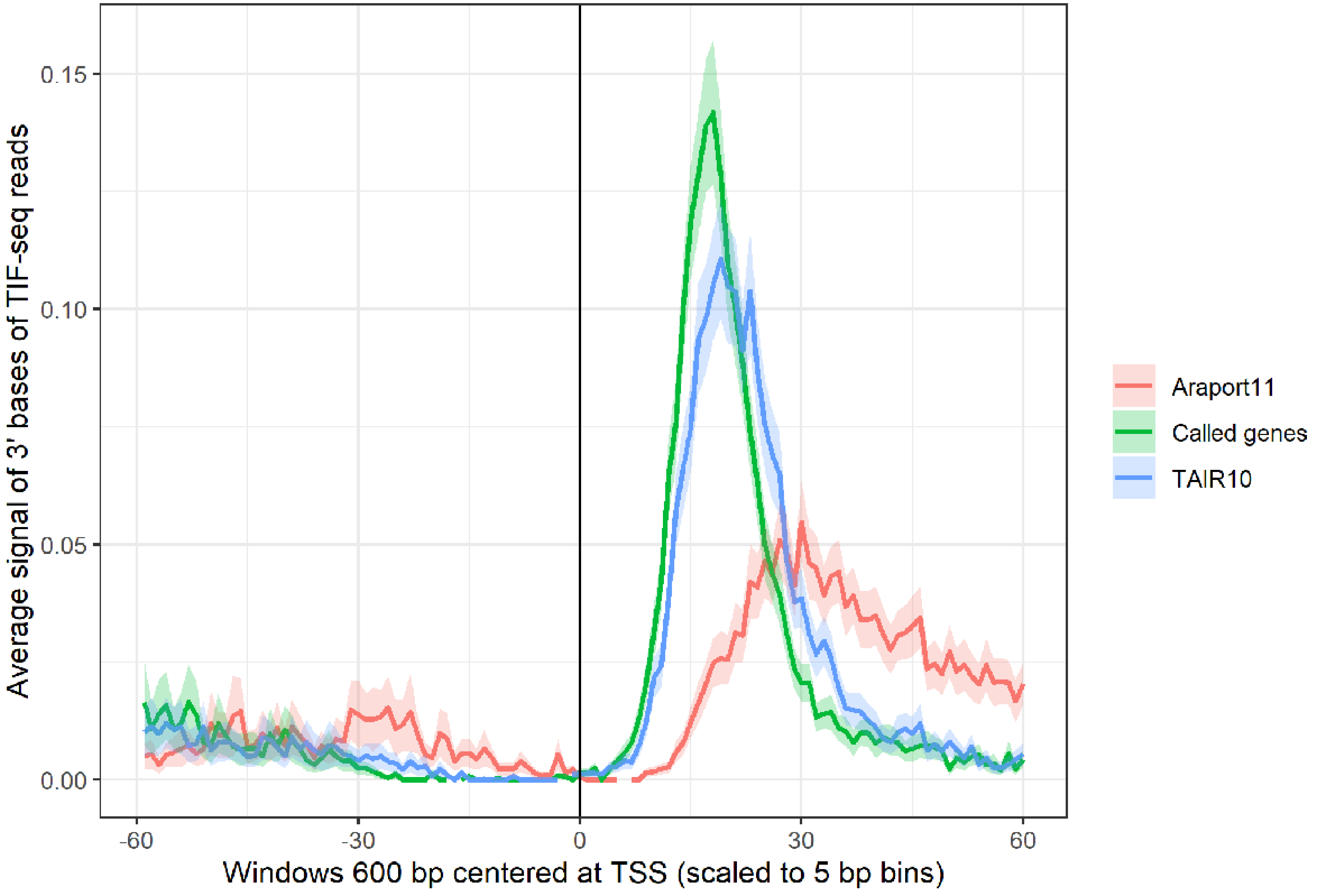

Average signal of 3' bases of TIF-seq reads
0.15
0.10
0.05
0.00
-60
-30
0
30
60
Windows 600 bp centered at TSS (scaled to 5 bp bins)
Araport11
Called genes
TAIR10

Figure S3

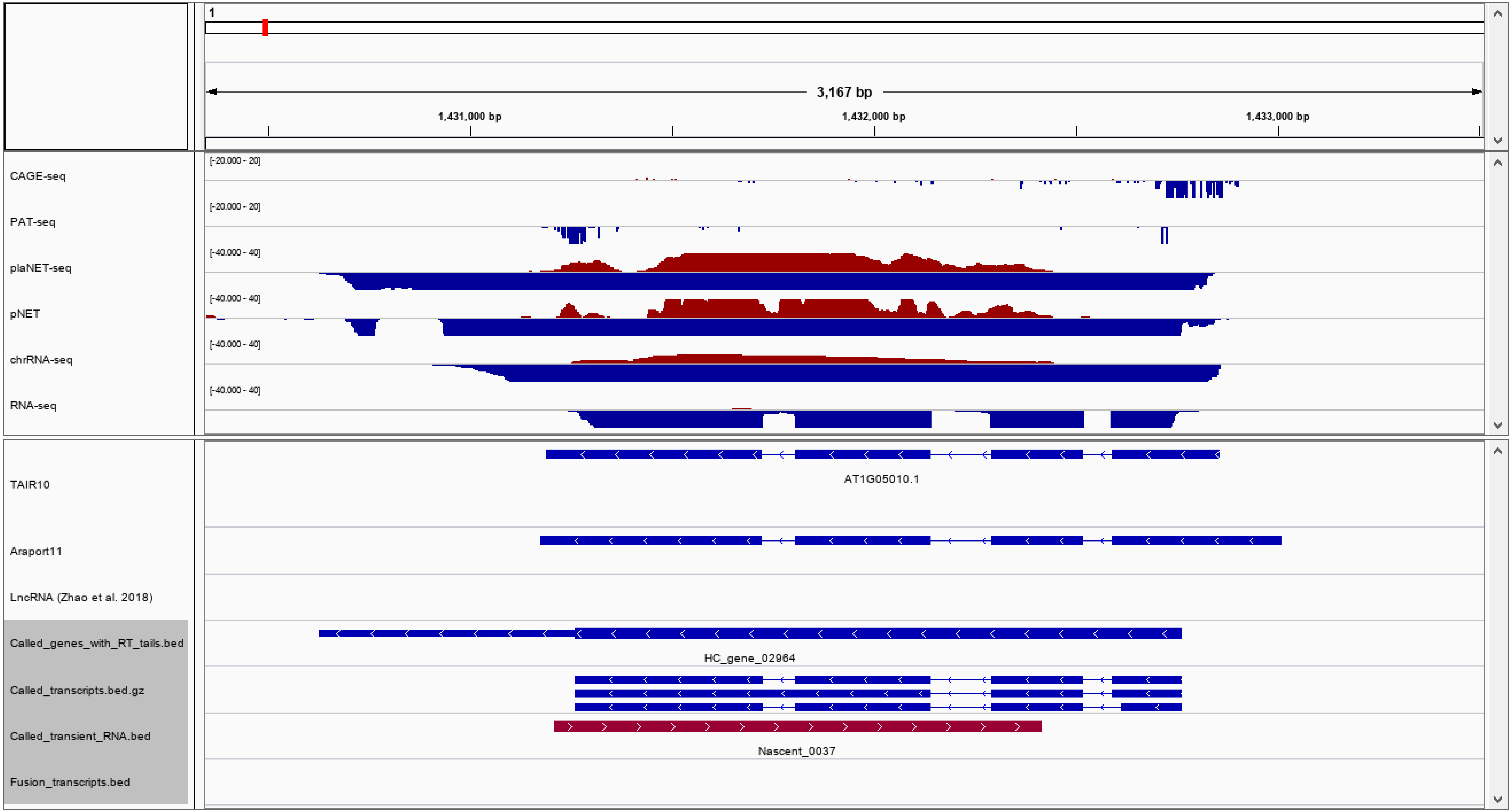

1
3,167 bp
1,431,000 bp
1,432,000 bp
1,433,000 bp
CAGE-seq
[-20.000 - 20]
PAT-seq
[-20.000 - 20]
plaNET-seq
[-40.000 - 40]
pNET
[-40.000 - 40]
chrRNA-seq
[-40.000 - 40]
RNA-seq
[-40.000 - 40]
TAIR10
AT1G05010.1
Araport11
LncRNA (Zhao et al. 2018)
Called_genes_with_RT_tails.bed
HC_gene_02964
Called_transcripts.bed.gz
Called_transient_RNA.bed
Nascent_0037
Fusion_transcripts.bed

Figure S4

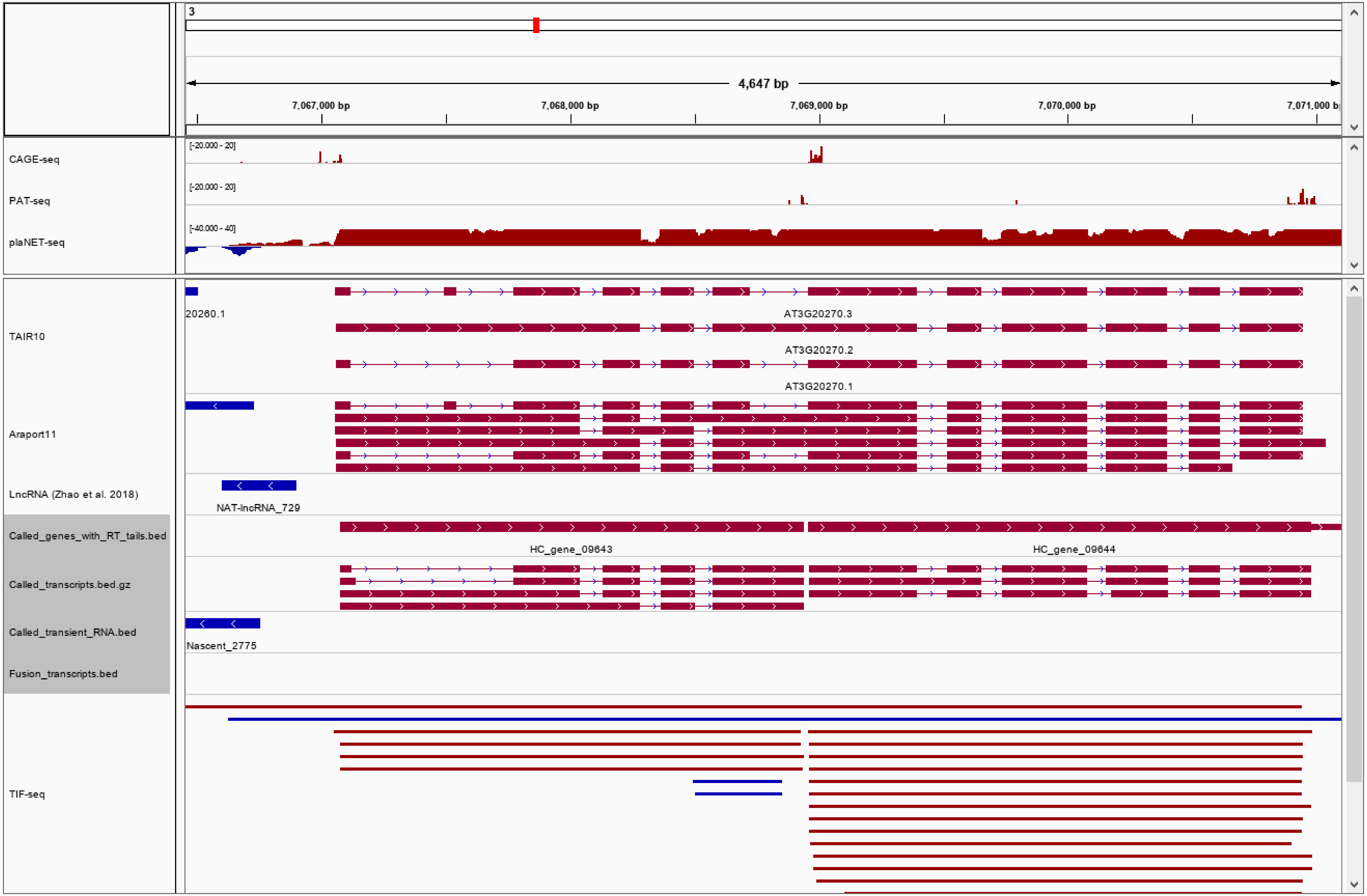

3
4,647 bp
7,067,000 bp
7,068,000 bp
7,069,000 bp
7,070,000 bp
7,071,000 b
CAGE-seq
[-20.000 - 20]
PAT-seq
[-20.000 - 20]
plaNET-seq
[-40.000 - 40]
TAIR10
20260.1
AT3G20270.3
AT3G20270.2
AT3G20270.1
Araport11
LncRNA (Zhao et al. 2018)
NAT-lncRNA_729
Called_genes_with_RT_tails.bed
HC_gene_09643
HC_gene_09644
Called_transcripts.bed.gz
Called_transient_RNA.bed
Nascent_2775
Fusion_transcripts.bed
TIF-seq
